# A Community-Based Framework Integrates Interspecific Interactions into Forest Genetic Conservation

**DOI:** 10.3390/plants13030435

**Published:** 2024-02-01

**Authors:** Xi Wang, Yu Xiao, Yan-Wen Lv, Zi-Han He, Francis C. Yeh, Xin-Sheng Hu

**Affiliations:** 1 College of Forestry and Landscape Architecture, South China Agricultural University, Guangzhou 510642, China; xwangaga@gmail.com (X.W.); yxiaoyu06@163.com (Y.X.); yanwenlvyx@126.com (Y.-W.L.); hezhan1717@163.com (Z.-H.H.); 2 Guangdong Key Laboratory for Innovative Development and Utilization of Forest Plant Germplasm, Guangzhou 510642, China; 3 Department of Renewable Resources, University of Alberta, 751 General Service Building, Edmonton, AB T6G 2H1, Canada; fyeh@ualberta.ca

**Keywords:** community structure, population structure, interspecific interaction, phylogenetic β-diversity, species coexistence

## Abstract

Forest genetic conservation is typically species-specific and does not integrate interspecific interaction and community structure. It mainly focuses on the theories of population and quantitative genetics. This approach depicts the intraspecific patterns of population genetic structure derived from genetic markers and the genetic differentiation of adaptive quantitative traits in provenance trials. However, it neglects possible interspecific interaction in natural forests and overlooks natural hybridization or subspeciation. We propose that the genetic diversity of a given species in a forest community is shaped by both intraspecific population and interspecific community evolutionary processes, and expand the traditional forest genetic conservation concept under the community ecology framework. We show that a community-specific phylogeny derived from molecular markers would allow us to explore the genetic mechanisms of a tree species interacting with other resident species. It would also facilitate the exploration of a species’ ecological role in forest community assembly and the taxonomic relationship of the species with other species specific to its resident forest community. Phylogenetic β-diversity would assess the similarities and differences of a tree species across communities regarding ecological function, the strength of selection pressure, and the nature and extent of its interaction with other species. Our forest genetic conservation proposal that integrates intraspecific population and interspecific community genetic variations is suitable for conserving a taxonomic species complex and maintaining its evolutionary potential in natural forests. This provides complementary information to conventional population and quantitative genetics-based conservation strategies.

## 1. Introduction

Forest genetic resources consist of primary and secondary gene pools [1]. The primary gene pools are the undisturbed natural forests, and the secondary gene pools are the remaining forests after ecosystem disturbance (including breeding populations and provenance trials). Many believe that genetic diversity in the primary gene pools can be conserved without the need to maintain it. However, as natural areas become progressively modified by human and non-human interferences, maintaining the genetic diversity of natural forests and their ecosystems will increasingly depend on the knowledge of intraspecific population and interspecific community evolutionary processes under the community ecology framework. A critical issue is that our current genetic conservation of a tree species lacks a community-oriented approach and overlooks the role of interspecific interaction in conservation.

A well-known fact is that forest trees are long-lived compared to crops, insects, and animal species. Trees often grow in heterogeneous habitats where the environmental conditions vary in time and space. They are sessile and are constantly under multi-year influences of the changes in the ecosystem and the pressure of either abiotic or biotic factors. The abiotic factors include natural environmental and physical habitats, such as rivers, mountains, climatic conditions, habitat fragmentation, and other barriers to species distribution. These factors interact with a tree species’ reproductive system (selfing, outcrossing, or a mixed system) to influence its population genetic structure [2]. Except for a small proportion of species that reproduce asexually only, most tree species have their geographic distributions formed by restrictive seed dispersal or pollen dispersal via pollination with recipient populations to produce seeds, followed by seedlings and subsequent growth. The abiotic factors impede seed and pollen dispersal and create population genetic differentiation or phylogeographical variation [3,4,5].

Biotic factors also influence species distribution, including intraspecific (e.g., density-dependent growth) and interspecific interactions [6,7]. A typical tree species distribution often covers diverse ecological communities and coexists with multiple species in its natural distribution. It may have positive, negative, or random associations with other species across communities. Such associations subsequently reduce genetic diversity if interspecific interactions produce directional selection acting on the tree species or maintain genetic diversity if balancing selection acting on the tree species is created.

In addition, natural hybridization and gene introgression frequently occur between closely related species in a forest community [8,9]. Hybridization occurs naturally in about 25% of plant species [10,11]. These hybrids contribute to species diversity, such as a hybrid zone as a barrier to isolating parental populations. Hybrids combined with recombination could generate transgressive segregation where extreme phenotypes that lead to speciation are formed [12,13]. These phenomena necessitate attention to hybrids in genetic conservation, which is not considered in species-based genetic conservation either.

Conventional forest genetic conservation is based on the theories of population and quantitative genetics [14,15,16,17]. Molecular markers are often applied to investigate population genetic structure ($F_{st}$) and the geographical pattern of genetic diversity and to infer potential routes of population formation and historical events [18,19,20,21]. Provenance trials, also known as common garden trials, are the planting experiments on multiple controlled field sites, with seedlings derived from seeds sampled from various locations in the natural distribution of a tree species [22]. Provenance trials are employed to investigate population genetic differentiation ($Q_{st}$, the same biological meaning as $F_{st}$) [23] and the geographical variation of quantitative traits, to delineate seed zones, and to develop seed allocation guidelines. These two pieces of information, one for population structure/history and the other for adaptive population differentiation, are then combined to determine the strategy of genetic conservation [4,24,25]. However, the results from both molecular markers and provenance trials do not provide information on species coexistence in natural forests. Provenance trials could examine site or genotype-by-environment effects and implicate conservation strategy in situ [26,27]. Because provenance trials are designed under controlled environmental conditions, the site effects imply the necessity of not protecting local habitats. Neither molecular marker-based nor provenance trials provide information on interspecific interactions and natural hybridization in natural forest communities.

The population genetic theory emphasizes the intraspecific interaction among genotypes, and its approach is widely applied to instructing genetic conservation [3,28]. The basic evolutionary processes that maintain genetic diversity involve selection, genetic drift, migration, and mutation at the population level [29]. These are naturally connected to the evolutionary processes that maintain biodiversity at the community level, including species selection, ecological drift, dispersal, and speciation [30,31,32,33]. These two levels of ecological and evolutionary processes not only participate in forest community assembly and succession but also shape the genetic diversity and evolution of tree species in the forest community. 

Community phylogeny effectively addresses interspecific interactions and natural hybridization, where community assembly can be characterized by lineage phylogenetic relationships [34]. Few reports are available to analyze community ecological functions and biodiversity conservation from the perspective of community phylogeny [35,36]. For forest genetic resource management, the analysis of forest community phylogeny also helps to determine the taxonomic status of a species in its community and its association with other species. A species may have the same or different taxonomical positions across forest communities, depending upon the community assembly and the phase of community succession [37,38,39]. Thus, it is interesting to integrate forest genetic conservation into the community ecology framework.

Here, we explore conserving forest genetic resources under this framework, which is rarely emphasized in previous works on forest genetic conservation. We begin by discussing the limitations of conventional species-based genetic conservation and its weakness that overlooks interspecific interactions and natural hybridization. We then discuss relevant theories, including community-specific phylogeny and the mechanisms of how a forest community shapes genetic diversity in natural forests. This provides a theoretical basis for the community-based genetic conservation of forest genetic resources. We finally discuss how to implement genetic conservation that integrates population and community processes in natural forests. This expands the conventional strategy of forest genetic conservation to community-based genetic conservation.

## 2. Limitations of Species-Based Genetic Conservation

Several limitations lie in the conventional species-based approach to genetic conservation. The first limitation comes from the potential taxonomic confusion, such as the lodgepole pine (*Pinus contorta*) and jack pine (*P. banksiana*) complex [8]. The well-known continuity of population evolution challenges researchers in conserving and utilizing genetic resources. One challenge is to determine a genetic conservation unit. A commonly accepted taxonomic classification system is the division that consists of species, genus, family, order, and so on. Species is a low-order taxonomic unit, but its definition remains complicated. Up to now, there are more than 20 species definitions [40], among which the biological species concept (BSC) and the phylogenetic species concept (PSC) are primarily used. BSC is a population of potentially breeding individuals reproductively isolated from similar natural populations [41]. PSC is the smallest diagnosable monophyletic group within which ancestor descendants coexist [42]. A commonality among various species concepts is that species differences are ultimately attributable to the long-term population genetic differentiation. Such population genetic differentiation provides the genetic basis for defining the concept of species in terms of reproductive isolation, morphological variation, lineage evolution, or ecological types [43].

Although subspecies and variety are not recognized in most species concepts, their introduction to some species makes it difficult to decide on genetic resource conservation. Subspecies and variety are mainly classified based on phenotypic and morphological traits. These traits are often affected by environmental factors and have low heritability. Genetic differences between subspecies or varieties could be slight [13]. For instance, *Toona ciliata* of the Meliaceae family, an endangered tree species in China, is considered to have five varieties according to the leaf and flower morphological traits [44]. Its genetic conservation is of increasing concern due to overcutting and a low natural regeneration rate in China. A conservation strategy based on the *T. ciliata* complex rather than a single variety was recommended from the analysis of the population genetic structure [3] and phylogenetic relationships among varieties [45]. A similar case was also reported in an endangered *Araucaria angustifolia*, whose varieties were rarely considered in conservation strategies [46,47]. Thus, subspecies or variety delimitation is necessary for some species to consider the interference of phenotypic variation due to incomplete lineage sorting. 

How to delimitate subspecies and variety remains challenging. Phylogenetic analysis helps to identify taxonomic positions of lineages and their evolutionary relationships. For instance, DNA barcodes are applied to identifying species by aligning target species sequences to the available database of reference DNA sequences [48]. The commonly used barcodes are the *rbcL* and *matK* sequences from the chloroplast genome, the ITS2 sequences from nuclear genomes, and the cytochrome oxidase I(COI) gene sequences from mitochondrial genomes [13]. However, restrictions in using DNA barcodes are the selection of appropriate DNA barcodes and the difficulty of identifying closely related species. It generally requires that barcodes exhibit little or no sequence variation within species but considerable variation between species or a small proportion of overlapping between intraspecific and interspecific variations. Natural hybridization and incomplete sorting make it challenging to select appropriate DNA barcodes. In addition, the barcodes from mitochondrial (rarely used in plant species) and chloroplast genes are uniparentally inherited and could differ from the barcodes from nuclear genomes (e.g., ITS2) in identifying species [49]. Thus, genome sequence analysis could help overcome this weakness and recognize varieties, subspecies, and closely related species [50].

The second limitation is the neglect of natural hybridization. The division of taxonomic units influences the strategy of conserving genetic resources [11,51]. One viewpoint is to use a management unit in conservation, which refers to the highly genetically differentiated populations where gene flow does not occur between populations [52]. Another viewpoint is to use the evolutionarily significant unit in conservation, which refers to monophyly regarding mtDNA variation or populations with significant population genetic differentiation in nuclear genome variation. Both units are based on population genetic differentiation, which often shows a contrasting pattern of population genetic structure, such as in the *T. ciliata* complex [3] and *Neolamarckia cadamba* [28]. Here, inconsistency could occur in delineating units regarding neutral molecular markers and adaptive phenotypic traits or in gene expression differences from transcriptome analysis [53]. This is because distinct evolutionary processes could produce genetic diversity among them. 

The taxonomic position of hybrids is controversial in conventional conservation. Hybrids are mistaken as rare species or not specified in either the 1992 Convention on Biological Diversity (CBD) adopted in Rio de Janeiro, Brazil, or in the Red List compiled by the International Union for Conservation of Nature and Natural Resources (IUCN) in 2010. It was the same in the U.S. Endangered Species Act (ESA) [11], where hybrids were not protected. Species-based conservation conceptually excludes hybrids in natural forests. Ennos et al. [54] argued that molecular markers can be used to elucidate the evolutionary process of forming taxonomic complexes and proposed to protect the evolutionary processes that maintain taxonomic biodiversity rather than single species. This approach covers the conservation of natural hybrids.

The third limitation is the neglect of interspecific interactions. Each species coexists with other species in a natural forest community. The species-based approach overlooks interspecific associations in conservation [55]. Population genetic theory on selection mainly refers to differential fitness among genotypes within species that come from intraspecific interactions. Although interspecific interaction has long been appreciated in population ecology, such as the classical Lotka–Volterra model of species competition [56,57], and recent empirical studies on interactions between *Picea mariana* and *Larix laricina* [58] or between pine tree and *Panax notoginseng* [59], it is generally excluded in population genetic theory and the practical management of forest genetic resources. 

## 3. Community Processes Shaping Species and Genetic Diversity

Before discussing genetic conservation under the community ecology framework, we examine how the evolutionary processes at the community level affect species diversity and the genetic diversity of a tree species at the population level. Here, we first describe the effects of community processes on species diversity, which helps to understand the role of a tree species and its interaction with other species in a forest community. We emphasize the interactions between trees and other plant species at the same trophic level. Interactions between trees and other organisms at different trophic levels, including birds, mammals, insects, and microorganisms, may also affect tree species diversity and genetic diversity in a forest community [60]. These effects are not concentrated. We then discuss the maintenance of genetic diversity under two levels (population and community) of evolutionary processes.

### 3.1. Community Processes Acting on Species Diversity

The succession of a forest community could influence the evolution of species diversity in the community [61]. There are four mechanisms for maintaining the forest community assembly: ecological drift, selection, speciation, and dispersal [31,32,62]. For instance, a community of small size generates a significant effect of ecological drift, which, on average, could yield more significant genetic drift effects for a species in the community. In a community of small size or a climax community, the genetic drift effects for some hyperabundant species could be slight. However, the average effects of genetic drift over all species in the community remain more extensive than those in communities of larger sizes.

Concerning the species coexistence in a forest community, various models of natural selection have been proposed [63], including the following:

- The stochastic niche or broken stick model [64], where the niche occupation and its size distribution of each species are random and independent of the niche sizes of other species in a community.
- The logarithmic normal distribution [65], where the niche size of a species is random and determined by the joint effects of many factors, and no selective advantage is present among species.
- The niche pre-emption model [66], where the first dominant species occupies the largest niche space, followed by the species that inhabits the second largest niche in the remaining space, to the last species that occupies the minimum niche.
- The neutral community model [31], where the community size is fixed, and other species equally compensate a decrease in one species’ abundance. All individuals in the community have similar birth and death rates.

When some lineages in a forest community are in the polyphyly or paraphyly phase or overlap in their ecological niches, species coexistence may occur in antagonistic or cooperative interaction [30]. For instance, the genetically close oak tree species are widely distributed to avoid resource competition [34]. In addition, a stochastic niche model may occur where species niches randomly change, reducing species diversity. The feedback between ecology and evolution could also shape the coexistence of species in a community. However, there are limited reports in the literature on species coexistence at the same trophic level [67,68].

Speciation is the ultimate source of species diversity in a forest community. Different speciation rates across communities can generate community differentiation [69]. Moreover, biogeographical processes (e.g., vicariance and dispersal) at a broader scale in both time and space also produce species diversity and the maintenance of distinct community structures across multiple habitats [70]. Similar to the role of speciation, species dispersal to a local forest community reduces inter-community differentiation in species diversity [37,71]. 

The above four evolutionary processes jointly maintain species diversity in a forest community and could produce distinct roles for a given species in assembling different communities. Speciation and dispersal increase species diversity in local communities, while selection and ecological drift often reduce species diversity. In time, an equilibrium between counter processes could be attained, giving rise to diverse communities specific to different habitats or environments.

### 3.2. Two Levels of Processes Acting on Genetic Diversity in a Forest Community

**Figure 1:**
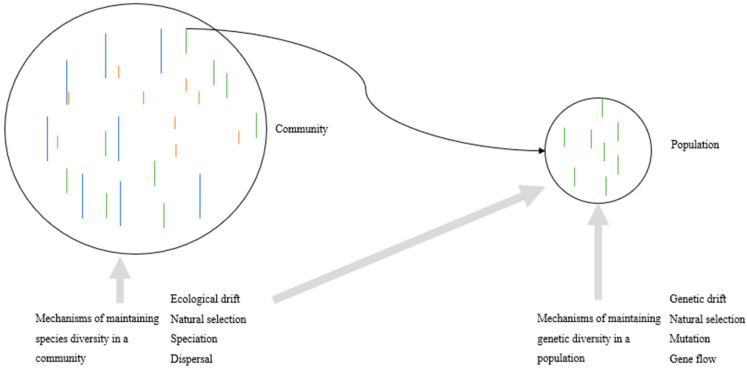
Two levels of evolutionary processes shape the genetic diversity of a species in a community. Different species are indicated in different colors in a community. Different individuals of the same species are indicated by the lines in the same color in a community or a population.

The population and community processes are not independent and jointly affect the spatial pattern of species genetic diversity in a forest community (Figure 1). Evidence supports that the genetic diversity of a species is positively correlated with the species diversity in its resident community, forming the so-called species–genetic diversity correlation (SGDC) [72,73,74,75]. Evidence supports a negative [76] or an independent SGDC [77,78]. The SGDC could vary with forest species and communities [63]. Schielzeth and Wolf [79] recently argued that population isolation, ecological niche, intraspecific selection, and neutral processes could produce a positive or negative SGDC. Thus, diverse patterns of SGDC in a forest community imply a complex relationship between the two levels of evolutionary processes in shaping the genetic diversity of a species. 

The specific expectations for the community processes to work on genetic diversity could be summarized below:

- Ecological drift simultaneously reduces selection efficacy at intraspecific and interspecific levels. This, on average, reduces the interspecific interactions and effective population sizes of individual species in a forest community. When the ecological drift effects (a small ecological community size *J*) increase, the role of a tree species in a forest community could change, depending upon the relative abundance of the species. If the population size of a tree species is smaller than the threshold size required for Allee effects to occur [80,81], the species is likely extinct. Suppose the population size is greater than the threshold size of Allee effects under the ecological drift effects. In that case, a smaller species population enhances the fixation probabilities of deleterious alleles, which weakens the adaptation of the species to its local habitats. Genetic diversity is expected to decrease.
- A forest community’s temporal succession could alter the role of individual species [82]. Some tree species would be lost, while others would dominate a forest community. Interspecific interaction directly affects the adaptation of a species and hence changes its genetic diversity. If a species abundance increases and gradually becomes the dominant species in a forest community, selection efficacy increases for adaptive alleles, and the species becomes more adaptive to the habitat. The genetic diversity at neutral loci may also increase in this species. However, if a species is disadvantageous during the forest community succession, the species may eventually be extinct, and so will its genetic diversity. Community assembly could evolve along the succession process, and the lineage phylogenetic relationship within the community also changes [83].
- Biogeographical processes, such as the invasion of alien species or new speciation, can change the status and adaptability of a tree species in a forest community [84]. Classical climate change also shapes forest community structure, and adaptive invasion species create competition with resident species in the recipient community and change species richness and abundance [85,86]. Inbreeding and/or outbreeding depression caused by invasive species could threaten the survival of local population genetic diversity. Maladaptive invasion subsequently changes its genetic diversity [87]. If a highly invasive species interacts with the tree species, this would substantially influence its genetic diversity. Effects of speciation on community structure could be observed when communities are investigated over a range of evolutionary, ecological, and geographic scales [88]. This is analogous to mutations that produce new genes and change the population’s genetic structure [29].

## 4. Characterizing a Forest Community in Terms of Phylogeny

Two fundamental properties of a forest community are related to the nature of the continuous evolution of populations and the artificial division in taxonomy. The first property is the phylogenetic relationship among tree species in a forest community. For instance, a phylogenetic tree among species can be re-constructed using orthologous single-copy gene sequences of multiple species in a forest community. According to the coalescent theory [89,90], orthologous genes among forest community species would eventually merge into common ancestors and form a gene tree. Evolution continuously proceeds at the population level and ultimately transforms the genetic variation within species into genetic divergence among species [91]. When a population is subdivided into multiple subpopulations and has reached a high level of genetic differentiation, the genetically differentiated subpopulations could gradually form new species, i.e., a shift from microevolution to macroevolution [92]. At the species level, the effects of evolutionary forces cause differential fitness among species and result in species selection. Although species selection is exceedingly slow [93], the process continuously proceeds. Some species might be eliminated due to their low competitive strengths, while others coexist despite interspecific interactions. Species coexistence may arise from stability where antagonistic intraspecific interactions are more significant than antagonistic interspecific interactions [30]. 

Selection and other processes could continuously transform the variation among species into genetic variation at a higher order. At the genus level, inter-group selection continues, and the “population” evolves to higher taxonomic units [94,95]. The lineages are connected through different phylogenetic relationships in a forest community and could form community-specific phylogeny among tree species. Because evolutionary processes are different across communities, the phylogeny could vary with different forest communities. Faith [96] proposed the concept of phylogenetic diversity to measure species diversity based on the phylogenetic tree. This analysis is now enhanced with genome sequence data.

The second property is that community-specific phylogeny helps to infer the events of natural hybridization, gene introgression, and lineage sorting for some specific species in a forest community. The continuity of population evolution could produce different phases of lineage sorting (polyphyly, paraphyly, and monophyly) for some tree species. Given some species divergent times, this could be inferred using genome sequence data [97]. Natural hybrids and gene introgression between genetically related species could be identified from phylogenetic analysis [98]. Also, potential hybridizations between specific species could be inferred from their branch groups in the community phylogeny. Although the field identification of hybrids is difficult from phenotypic traits, the hybridization could be inferred from genetic markers. A tree species may have different evolutionary branch lengths in different community phylogenies. All these analyses provide vital information for developing the strategy to conserve the genetic resources of a tree species, which is only possible to derive with community phylogenetic analysis.

The preceding discussions are conducted from a more fundamental perspective, complementing previous reviews on community phylogeny [36,99]. A forest community can be characterized by a phylogeny, which is essentially related to community richness. Information on species abundance could implicitly be derived from phylogeny, assuming that older species are expected to be more abundant than young species [36]. This requires accurate estimates of date nodes for the ancestral species/populations in phylogeny. With managed or intensively managed forest communities, the distribution of species abundances could be changed compared to natural stands in species richness and diversity [100,101]. Under this situation, phylogenies may differ in branch length and topological structure between managed and natural communities when species richness, especially that of the dominant species, is changed. Whether the phylogeny-based approach is better than a conventional β-diversity-based approach to describe species abundance remains to be determined.

## 5. Community-Based Strategy of Genetic Conservation

We now discuss how information on community structure can be applied to the genetic conservation of a forest species. Three steps are necessary to implement community-based genetic conservation. The first step is to determine the number of communities that deserve conservation. The second step is to detect interspecific interaction. The third step is integrating the population and community levels of evolutionary processes into genetic conservation.

### 5.1. Determining the Number of Communities

We use β-diversity to characterize the degree of community differentiation in species assembly. This index can be calculated differently [102,103,104,105]. Many studies have used β-diversity to describe biodiversity [106,107,108]. However, few studies have associated the community evolutionary processes with β-diversity. This line of work remains in its infancy but is of significance to better understand the mechanisms that maintain community differentiation [109]. A straightforward case is under the neutrality assumption where the community size is fixed (*J*) and other species equally compensate a decrease in one species’ abundance. All individuals in the community have the same birth and death rates [31]. Suppose that there are an infinite number of local communities each with *J* individuals, analogous to Wright’s island model in population genetics [29]. Community differentiation ($C_{st}$) is derived below under an equilibrium of ecological drift (1/J) in any local community, the dispersal rate (m) to each local community, and the speciation rate (v) [71]:

(1) $$C_{st}=\frac{1}{1+2J\left(m+v\right)}$$

Dispersal across communities and speciation reduce β-diversity ($C_{st}$) and tend to homogenize community assembly or community phylogenies. This relation may be used as a null hypothesis to test whether the selection process is broadly engaged in community differentiation.

With the conservation of forest genetic resources, β-diversity is used to design a network for protected communities. When a large proportion of the community differentiation (e.g., 95%) occurs, the community difference in species composition could be substantial. The protected area should cover multiple communities and more species. Heterogeneity in interspecific interaction could occur for a tree species with other species across the communities. In contrast, when there is a low level of community differentiation (e.g., less than 5%), only a few communities should be selected for conserving species diversity. This is analogous to the decision making on conserving genetic diversity based on $F_{st}$ value for a single species [18,24]. A decision based on β-diversity focuses on the tree species diversity at the community level.

**Figure 2:**
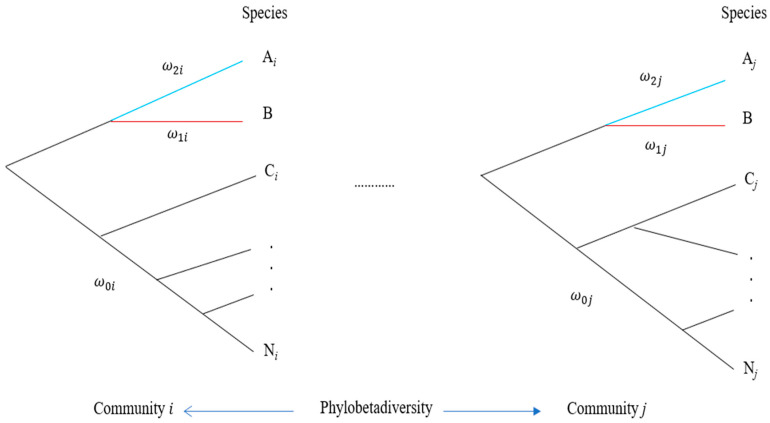
Determination of the number of communities based on the pattern of phylobetadiversity. A species of interest (species B) is indicated in red under hypothetical community phylogenies (communities *i* and *j*). Letters A*_i_*, C*_i_*, …, and N*_i_* represent different species in community i. Letters A*_j_*, C*_j_*, …, and N*_j_* represent different species in community j. All ω values on different branches can be estimated under different hypotheses of branch models in a community.

Apart from characterizing community differentiation by β-diversity, a further measurement is to combine β-diversity with phylogeny to define phylobetadiversity that measures community differentiation in terms of both community phylogeny and β-diversity (Figure 2). This index is calculated by the distance (branch length differences) between phylogenetic trees of different communities. Several methods have been proposed to estimate this index. Graham and Fine [110] discussed the application of phylobetadiversity in a biodiversity study, including an analysis of speciation, a combination with niche models, neutral theory, and global biodiversity patterns. However, the practical application of phylobetadiversity to conservation is rarely emphasized in the literature [111]. In theory, phylobetadiversity can also be used to design the number of communities for conservation, analogous to the use of β-diversity. A slight difference in phylobetadiversity implies that a few communities are appropriate for conservation.

Compared with β-diversity, the analysis of community phylobetadiversity provides two additional pieces of information to guide genetic conservation: (1) Test the selection pressure experienced by a tree species in different communities (see the following subsection), including its subspecies and potential hybrids. This is a phylogeny-based method not provided by conventional β-diversity analysis. To measure the strength of natural selection, we apply ω = $k_{a}⁄K_{s}$ value, meaning the ratio of nonsynonymous substitutions to synonymous substitutions. Based on the community-specific phylogeny, we can estimate ω on the branch of the tree species according to the branch model or ω at the amino acid sites of genes of the tree species according to the site model [112]. The type of selection is then indicated: ω > 1 for positive selection, ω = 1 for neutrality, and ω < 1 for purifying selection. We can compare the relative intensities of natural selection of a specie and its interacted species across forest communities. This also helps to infer the reasons for generating differences in genetic diversity in different communities. For instance, a difference may arise from distinct interspecific interactions of a given species with other species or random genetic drift. Evidence supports the spatial variation of species fitness due to environmental factors [113]. (2) Analyze the status differences of a given tree species among communities. This may include the taxonomic and evolutionary positions and the branch lengths in the phylogenies of different communities. This is also not provided by conventional β-diversity analysis. By combining the species abundance distribution across forest communities, we can analyze the role of a tree species in community assembly and ecological functions. Note that the objective of determining the number of communities is for detecting the interspecific interaction of a species in the second step. This helps to reduce cost and conserve the appropriate genetic variation of a species across communities. 

The practical analysis of community differentiation needs setting up forest plots, such as the forest census plots in Barro Colorado Island [114] and multiple plots in China [115,116], to survey species richness and abundance for a given community. The ideal case is that entire plots are surveyed once a community boundary is set, but this forest census requires a high cost. Instead, multiple quadrats are often designed for forest surveys in each community. Community differentiation is then measured using some beta-diversity indices. Moreover, to identify hybrids of a species with other related species, large samples, such as more plots or quadrats, are preferred to improve the probability of capturing hybrids.

Accompanying community surveys by setting multiple plots or quadrats are the population samples of a species of interest. When the species is abundant in each community, a large sample size of the species per community, for example, >30 individuals, is recommended. When the species is less abundant in some communities, more quadrats are suggested to include the species as much as possible to assess the species’ population structure appropriately. 

As mentioned above, the careful selection of orthologous genes or barcodes is needed to genotype all sampled individuals. High polymorphic markers, such as nuclear ITS markers, are recommended to identify hybrids or closely related subspecies. Chloroplast or mitochondrial DNA markers are appropriate to elucidate community phylogenetic relationships among distant species. Multiple orthologous nuclear gene sequences are relevant to examining genetic variation within and between species.

### 5.2. Detecting the Molecular Mechanism of Interspecific Interactions

**Figure 3:**
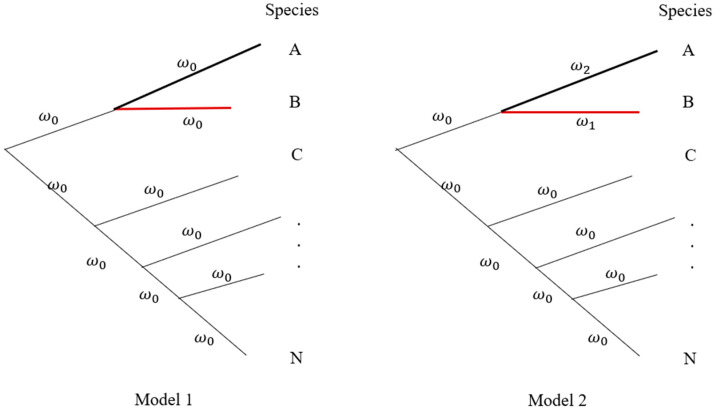
A hypothetical phylogeny in a forest community. Different species may have unequal branch lengths. Model 1 assumes that all branches have equal selection strength ($\omega_{0}$). Model 2 assumes that species A ($\omega_{2}$) is closely related to Species B ($\omega_{1}$) under different selection intensities. All remaining species are set to have the same selection intensity ($\omega_{0}$). Letters A, B, C, …, and N represent different species in a forest community.

The second step is to screen interspecific interaction for tree species in different forest communities. Various methods have been proposed to test species associations [117], such as the method based on species presence/absence data among quadrats [118] or the chi-square test using contingency tables [119]. Cavender-Bares et al. [34] reviewed species competitive interaction as a mechanism for phylogenetic relatedness. Here, we concentrate on the question of how to detect molecular mechanisms underlying interspecific interactions. Using multiple orthologous gene sequences among species in a community, we can construct a community-specific phylogenetic tree. We then design different hypotheses to test selection on the branch of a given tree species. For instance, a branch model may be set to test the relative selection pressure for the interspecific species in a forest community. Figure 3 shows a hypothetical phylogenetic tree of a forest community. Suppose species B is the tree species of interest, and species A is its closely related species with interaction. We may set the branch model in the PAML (phylogenetic analysis by maximum likelihood) program [120] to detect ω ($=K_{a}⁄K_{s}$) and evaluate the relative selection pressure on different branches. For instance, two models are assumed in Figure 3. The logarithm of likelihood ratio test (LRT), $-2\ln\left(\frac{l_{2}\left(\omega_{1},\omega_{2},\ldots ,\omega_{0}\right)}{l_{1}\left(\omega_{0},\omega_{0},\ldots ,\omega_{0}\right)}\right)\sim {Χ}^{2}\left(df=2\right)$, is applied to test whether the interacted species have significantly natural selection or not. With the analyses of multiple ecological communities, the change of ω on the branch of the target tree species signals different selection pressures or the difference of the target tree species interacting with different species across communities.

Besides the branch model, site or branch–site model analysis may also detect the positive or purifying selection of specific genes at the amino acid sites. This helps to screen particular genes involved in interspecific interaction in different communities.

It is essential to understand that disruptive selection facilitates speciation where alternative alleles tend to be fixed in different species. However, incompletely sorted lineages tend to occupy similar ecological niches and result in competition. This could vary in various communities. One prediction is that ecological drift (1/J) reduces the efficacy of species selection and weakens interspecific interaction in a small forest community. However, this benefits the species vulnerable to survival, which can be analogously implied from the population genetics theory [121,122,123,124]. Consider a diploid nuclear gene in a population with effective population size $N_{e}$. Under an equilibrium of selection and genetic drift effects, Kimura [122] derived the fixation probability of a mutant allele. When the mutant allele is favorable (positive selection), the ratio of nonsynonymous to synonymous divergence among orthologous genes is

(2) $$\frac{K_{a}}{K_{s}}=\frac{4N_{e}s}{1-e^{-4N_{e}s}}$$

(3) $$\frac{\partial (K_{a}/K_{s})}{\partial N_{e}}=\frac{4se^{-4N_{e}s}\left(e^{4N_{e}s}-1-4N_{e}s\right)}{{\left(1-e^{-4N_{e}s}\right)}^{2}}>0$$

The product of the effective population size and selection coefficient, $\;N_{e}s$, can be viewed as a scaled-selection coefficient and measures the strength of natural selection. An analogous expression to $k_{a}⁄K_{s}$ for the fixation of a species relative to that under a neutral community in a local community of size *J* is not available yet. It is speculated that a large forest community could tend to facilitate the species with synergistic interacted effects (e.g., mutualism) on a species and improve species selection efficacy. A small forest community could impede such synergistic effects. However, population genetic theory implications need further theoretical confirmation in community ecology.

When the mutant allele is deleterious (purifying selection) in a population, the ratio of nonsynonymous to synonymous divergence is

(4) $$\frac{K_{a}}{K_{s}}=\frac{-4N_{e}s}{1-e^{4N_{e}s}}$$

(5) $$\frac{\partial (K_{a}/K_{s})}{\partial N_{e}}=\frac{-2s\left(1-e^{4N_{e}s}+4N_{e}se^{4N_{e}s}\right)}{{\left(1-e^{4N_{e}s}\right)}^{2}}<0$$

Similarly, it is speculated that a small forest community could weaken the purifying selection of the target tree species, while a large forest community could strengthen the purifying selection. This could likely weaken the antagonistic interacted effects on a species. Empirical evidence implicitly supports this theoretical prediction under communities of different sizes. Kapralov [125] used 36 orthologous nuclear genes to estimate the $K_{a}/K_{s}$ ratio of 27 species of the genus *Schiedea* (Caryophyllaceae) sampled from the Hawaiian Islands and the mainland. The results showed that the $K_{a}/K_{s}$ values were higher in the island group than in the mainland group. This was because the purifying selection in the island group was relaxed, and positive selection was more common than in the mainland group. Therefore, it is hypothesized that a tree species could undergo different intensities of natural selection in communities of different sizes. More evidence is needed to verify this prediction.

### 5.3. Integrating Two Levels of Evolutionary Processes into Genetic Conservation

The third step is integrating information from population genetic structure, community structure, and interspecific interactions into genetic conservation. Under the community framework, if the sampling sites are set in different forest communities, both community phylogeny and interspecific interactions help to interpret population genetic differentiation. When the effects of interspecific interactions are more significant than the effects of genetic drift, the following outcomes could be yielded:

- When the given tree species has different patterns of selection across communities, e.g., a half number of communities with positive selection ($K_{a}/K_{s}>1$) and another half with purifying selection ($K_{a}/K_{s}<1$), the effects of interspecific interactions can amplify population genetic differentiation. Population genetic differentiation is more significant than that under neutrality. Selection due to interspecific interactions would increase the genetic differentiation of the species at the population level, which is analogous to the outcome of genetic hitchhiking effects or background selection effects on genetic differentiation at a linked neutral site [126,127,128].
- When the given tree species has the same degree and pattern of interspecific interactions across communities, e.g., all communities with a similar extent of purifying/positive selection, population genetic differentiation may be smaller than that under neutrality.
- When the given tree species has different extents and types of interspecific interactions in different communities, such as weak positive and purifying selection, population genetic differentiation may be close to that under neutrality. An appropriate number of community-based populations could be suggested for conservation from this array of patterns of the population genetic differentiation of a given species.

When interspecific interactions are ignorable, our suggested strategy reduces to the conventional $F_{st}$ and $Q_{st}$ schemes that rely on population and quantitative genetics theories [15,25,129].

One caution is that when the ecological drift effects are unequal and highly fluctuate across communities, a given tree species’ effective population size ($N_{e}$) may also be different among communities. The ecological drift (1/J) strengthens the genetic drift and amplifies population genetic differentiation. Other processes, such as species invasion or distinct speciation rate, could influence the genetic differentiation of a given tree species through species interactions [71]. Therefore, it is necessary to consider the type of forest community, phylogeny, and interspecific interactions in genetic resource conservation, which provides complementary information to the conventional ($F_{st}$)-based genetic conservation. 

In summary, with the community-based strategy of genetic conservation, the idea of biotope protection proposed by Ennos et al. [54,130] can be realized. We can determine an appropriate number of protected communities according to the information on β-diversity and phylogenetic β-diversity. The hybrids and subspecies for some species can be protected in single or multiple communities. A strategy for conserving genetic diversity can be created by combining the spatial distribution of genetic diversity and genetic differentiation in protected communities. A comprehensive analysis of the patterns at two levels can determine the number of communities and populations of a given tree species. We can also reveal the status of a species, its relatives, and potential hybrids in a forest community from community-specific phylogeny. When a forest community is protected, a species and its hybrids or the variety of some species are also conserved. Biotope protection naturally conserves the evolution potential of a given tree species and its interaction with other species. This makes up for the weakness of individual species-based strategies of genetic conservation.

## 6. Conclusions

A long generation cycle, high heterozygosity, high genetic loads, sessility, and growth in heterogeneous habitats characterize forest trees. In the long-term evolution process, many mutants have accumulated and provided rich materials for selection and utilization. The traditional conservation of forest genetic resources is mainly based on the theory of population and quantitative genetics, with a single species as the unit. From the information on the population structure of both adaptive quantitative traits and molecular markers, an appropriate strategy is formulated for the genetic conservation of a forest species. The limitation of this approach is that it neglects the following:

- The interactions of the given tree species with other species in natural forests, which affect genetic diversity.
- The conservation of potential natural hybrids between a species and its closely related species.
- The conservation of the genetic diversity of varieties or subspecies of some species.

We explore the strategy to conserve forest genetic resources under the community ecology framework. The genetic diversity of a tree species is not only controlled by the evolutionary forces of genetic drift, individual selection, mutation, and migration at the population level but also by the evolutionary forces of ecological drift, species selection, speciation, and dispersal at the community level. The analysis of community-specific phylogeny helps to reveal the mechanism of interspecific interactions, the role of a given tree species in forest community assembly, the lineage relationships of the tree species with its relatives, including potential hybrids, and the effects of interspecific interactions on the genetic diversity of the species. The analysis of phylogenetic β-diversity reveals the difference in ecological status, selective pressure, and interspecific interactions of a given species with other species in different communities and the effects of the two levels of evolutionary forces on population genetic differentiation (*F_st_*). This information helps to determine the number of communities and populations, which makes up for the limitations of conventional genetic conservation based on a single species only.

## Data Availability

Not applicable.
